# Higher rates of non-skeletal complications and greater healthcare needs in achondroplasia compared to the general UK population: a matched cohort study using the CPRD database

**DOI:** 10.1186/s13023-023-02811-5

**Published:** 2023-07-25

**Authors:** Jeanne M. Pimenta, Melita Irving, Moira Cheung, Louise Mazzeo, Sarah Landis, Swati Mukherjee

**Affiliations:** 1 BioMarin (UK) Limited, London, UK; 2 Guy’s and St Thomas’ NHS Foundation Trust, London, UK

**Keywords:** Achondroplasia, Clinical Practice Research Database, CPRD, Hospital Episode Statistics, HES, Matched cohort study

## Abstract

**Background:**

The natural history of skeletal complications in achondroplasia (ACH) is well-described. However, it remains unclear how the rates of non-skeletal complications, surgical procedures, healthcare needs and mortality differ between individuals with ACH and the general population. This study aimed to contextualise the extent of these outcomes by comparing event rates across the lifespan, between those with ACH and matched controls in a United Kingdom (UK) population.

**Methods:**

This retrospective, matched cohort study used data from national UK databases: the Clinical Practice Research Database (CPRD) GOLD from primary care, the secondary care Hospital Episode Statistics (HES) databases and the Office of National Statistics mortality records. ACH cases were identified using disorder-specific Read Codes or International Classification of Diseases 10th Revision codes. For each ACH case, up to four age- and sex-matched controls (defined as those without evidence of skeletal/growth disorders) were included. Event rates per 100 person-years were calculated for a pre-defined set of complications (informed by reviews of existing ACH literature and discussion with clinical authors), healthcare visits and mortality. Rate ratios (RRs) with 95% confidence intervals (CIs) were used to compare case and control cohorts.

**Results:**

541 ACH cases and 2052 controls were identified for the CPRD cohort; of these, 275 cases and 1064 matched controls had linkage to HES data. Approximately twice as many non-skeletal complications were reported among individuals with ACH versus controls (RR [95% CI] 1.80 [1.59–2.03]). Among ACH cases, a U-shaped distribution of complications was observed across age groups, whereby the highest complication rates occurred at < 11 and > 60 years of age. Individuals with ACH had greater needs for medication, GP referrals to specialist care, medical imaging, surgical procedures and healthcare visits versus controls, as well as a mortality rate of almost twice as high.

**Conclusions:**

Patients with ACH experience high rates of a range of both skeletal and non-skeletal complications across their lifespan. To manage these complications, individuals with ACH have significantly increased healthcare needs compared to the general population. These results underscore the need for more coordinated and multidisciplinary management of people with ACH to improve health outcomes across the lifespan.

**Supplementary Information:**

The online version contains supplementary material available at 10.1186/s13023-023-02811-5.

## Introduction

Achondroplasia (ACH) is an autosomal dominant skeletal dysplasia, and the most common form of disproportionate short stature[1]. It is rare, with an estimated worldwide and European prevalence of 4.6 and 3.5 per 100,000 live births, respectively [2], translating to more than 250,000 affected individuals worldwide [3].

ACH is caused by a recurrent dominant pathogenic variant within the fibroblast growth factor receptor 3 gene (*FGFR3*) [1, 3], which leads to the inhibition of endochondral bone growth [4]. As a consequence of this, individuals with ACH have disproportionate short stature, rhizomelia, macrocephaly with frontal bossing, midface hypoplasia and a range of other serious skeletal complications. Individuals can also experience a range of serious and debilitating multisystem complications [3–8]. A particularly severe manifestation occurring during infancy and early childhood is foramen magnum stenosis, which can cause compression of the spinal cord at the craniocervical junction, risking sudden death [4, 9]. Skeletal complications including spinal stenosis, abnormal curvatures in the spine, joint contractures and lower limb deformities impact individuals at a range of ages [3, 4, 10, 11], and lead to reduced quality of life across physical, social, emotional and school/work functioning aspects [12, 13].

As a predominantly skeletal condition, there has been a greater focus on the understanding and management of skeletal complications of ACH, with less attention paid to qualifying and quantifying non-skeletal effects. In addition, while the natural history of ACH is relatively well-described in the paediatric age group, there is much less information on the progression of ACH in adults and the impact this has on surgical procedures and healthcare needs [14]. Finally, whilst studies have found that the rate of early mortality among individuals with ACH are increased compared with the general population in the United States (US) [14–17], mortality rates for individuals with ACH outside this region remain to be elucidated.

This study aimed to contextualise the extent of ACH complications, healthcare needs and mortality within the United Kingdom (UK) ACH population by comparing the rates of these events among individuals with ACH to those among matched controls from the general UK population.

## Methods

### Study design and data sources

This retrospective, observational, matched cohort study used data from the UK Clinical Practice Research Database (CPRD) GOLD, a research service providing anonymised electronic health records (EHRs) from general practitioner (GP) practices across the UK since 1987. CPRD data were supplemented with secondary care data from Hospital Episode Statistics (HES) and mortality records from the Office of National Statistics (ONS).

The CPRD is a real-world database providing a quality-assured and validated source of longitudinal and representative UK population primary healthcare data for epidemiologic research. Collecting anonymised EHR data since 1987, the CPRD GOLD database includes records from over 60 million patients, with 16 million currently registered patients [18]. The EHR includes a wide range of data including patient demographics, clinical events, prescription details, referrals to specialists and hospital admissions, in addition to laboratory results, lifestyle details and height/weight (as captured by GPs using the Vision^®^ patient management system).

This study used several sources of secondary care data linked to the CPRD data. The HES Admitted Patient Care (HES APC) dataset contains details of episodes of care delivered by (or on behalf of) the National Health Service (NHS) to patients admitted to hospitals in England. HES APC was used to determine in-patient experience, and contains data including patient demographics, clinical information on diagnoses, operations/procedures, and dates of admissions and discharge. The Hospital Outpatient Data (HES OP) dataset was used as a source of outpatient appointment data, and the HES Diagnostic Imaging Dataset (HES DID) for information on type and body site of diagnostic imaging tests. Furthermore, death information, including date and cause of death, were provided via linkage of HES data to death registration data from the ONS. Linkage between datasets was undertaken by NHS Digital which acts as a ‘trusted third party’ for CPRD and holds the linkage identifiers for all datasets.

Full details on all datasets and linkage methodology, as well as the criteria used to determine whether patient data were acceptable for inclusion in the study, are provided in Additional file 1.

### Study population

This study compared data from individuals with ACH (‘cases’) with those from the general UK population (‘controls’). A cohort of cases with ACH was identified using database-appropriate diagnostic codes; disorder-specific Read Codes within the CPRD database and a disorder-non-specific International Classification of Diseases 10th Revision (ICD-10) code within the HES database. An iterative algorithm was applied to identify definitive ACH cases, and exclude other dysplasias, specifically hypochondroplasia and pseudoachondroplasia from the ACH cohort. Full details of the methodology used to identify cases are included in Additional file 2. A pool of potential controls was defined as those individuals in the databases with no previous record of ACH, other types of dwarfism, or conditions affecting stature (Additional file 3). These were the only exclusion criteria applied.

The study period ran from 1st January 1987 to 31st December 2018. Patient follow-up began on the study start date or if the patient entered the database at a later date, on the patient’s database registration date. Follow-up ended on the date of patient transfer (patient moving to another GP outside the database or end date within the database), patient death or on 31st December 2018, whichever occurred first.

### Cohort matching

Up to four controls from the pool of potential controls were matched to each individual with ACH based on GP practice, year of birth (± 1 year), sex and linkage eligibility to HES, using index date matching (see Additional file 2 for full details). The index date for ACH cases was defined as the first record of ACH in the CPRD or HES APC database during the study period.

Analyses were conducted in two study cohorts. The larger primary care cohort consisted of all eligible patients from CPRD GOLD and included patients across the UK (‘CPRD cohort’). The second cohort consisted of patients from the CPRD GOLD who were eligible for linkage to HES data, providing linked primary and secondary care (‘CPRD HES-linked cohort’); this cohort therefore used data from England only.

### Outcomes and analyses

Study outcomes included the type and rate of skeletal and non-skeletal complications, medication use, GP referrals to specialist care, surgical procedures, healthcare visits and medical imaging procedures, as well as the rate and cause of mortality. Analyses of complications, medication use and GP referrals to specialist care were based on the CPRD cohort only, while analyses of surgical procedures, healthcare visits, medical imaging procedures and mortality were conducted for the CPRD HES-linked cohort, as the linked dataset represented the most complete data for these outcomes.

The study focused on a pre-defined set of complications and associated surgeries, which had been reported within the ACH literature. For skeletal complications (defined using Read codes in CPRD and ICD-10 codes in HES data) and surgeries (defined using Operating Procedure Codes Supplement [OPCS] codes), the pre-defined list was informed by reviews of existing ACH literature and discussion with clinical authors [4, 19–21]. For non-skeletal complications and surgeries, a broader set of pre-defined categories, grouped according to body systems, was used, given the lack of existing literature regarding non-skeletal complications in ACH. Medications and medical imaging were also based on broader categories and grouped according to the body systems of the associated complications. Within the CPRD dataset, the specific indication for medication use is not recorded and some medications may be used for multiple, different indications. For the purposes of this study, the primary indication for medication use was selected. GP referrals to specialist care were not based on a pre-defined list; all referral types were assessed. For mortality, in addition to number and rate of death, the underlying cause of death (defined by ICD-10 chapter) was assessed.

In this descriptive study, event rates (ERs) per 100 person-years (100PY) were calculated, using the total number of events divided by total person time, for events among the case and control populations, as well as by age group for ACH cases only to understand age-related trends of events in this population. For complications and surgical procedures, occurrences of the same event that were reported within 30 days of each other were considered to be the same episode and so only counted as a single event. To facilitate comparisons between cases and controls, rate ratios (RRs) were calculated by dividing the event rate of a given outcome amongst ACH cases by that amongst matched controls. Corresponding 95% confidence intervals (CIs) were also calculated, taking into account the matching. For healthcare visits, the mean (standard deviation [SD]) number of annual visits among cases and controls were calculated and compared using two-sided t-tests [22].

Due to CPRD reporting requirements, to maintain patient confidentiality, instances in which fewer than five cases or controls experienced the outcome are not permitted to be reported as an exact number; in such cases, data were presented as ‘ < 5’. All analyses were performed using SAS version 9.4 (SAS Institute Inc).

### Informed consent and ethical approval

This study used the CPRD and HES databases of pseudonymised patient EHRs; therefore, patients’ informed consent was not required for this study. CPRD Independent Scientific Advisory Committee (ISAC) approval for this study was obtained on 29th August 2018 (application number: 18_189RA).

## Results

### Patient characteristics and follow-up

A total of 541 ACH cases and 2052 controls were identified for the CPRD cohort. Of these, 275 ACH cases and 1064 controls had linkage to HES data. Patient characteristics for cases and controls are presented in Table 1. As expected, the variables used in matching (the gender, location and age) were very similar between cases and controls; however, the mean overall follow-up time was slightly longer for controls as compared to cases. Patient characteristics, with the exception of region, were also similar between the CPRD and CPRD HES-linked cohorts. The majority of participants (68%) within the CPRD cohort were from England, with smaller proportions located in Scotland (16%), Wales (14%) and Northern Ireland (2%). There was a broad representation of regions across England in both the CPRD and CPRD HES-linked cohorts.

**Table 1:** Baseline characteristics of individuals with ACH and controls (CPRD and CPRD HES-linked cohorts)

Characteristic	CPRD		CPRD HES-linked	
	Cases (N = 541) n (%)	Controls (N = 2052) n (%)	Cases (N = 275) n (%)	Controls (N = 1064) n (%)
*Gender*				
Male	264 (49)	1001 (49)	136 (49)	526 (49)
Female	277 (51)	1051 (51)	139 (51)	538 (51)
*Location*				
England	366 (68)	1394 (68)	275 (100)	1064 (100)
East of England	62 (11)	225 (11)	48 (17)	182 (17)
South Central	50 (9)	195 (10)	36 (13)	140 (13)
London	50 (9)	188 (9)	35 (13)	132 (12)
North West	49 (9)	190 (9)	39 (14)	152 (14)
South West	40 (7)	156 (8)	36 (13)	144 (14)
West Midlands	40 (7)	154 (8)	24 (9)	93 (9)
South East Coast	33 (6)	126 (6)	27 (10)	103 (10)
Yorkshire and The Humber	16 (3)	59 (3)	12 (4)	47 (4)
East Midlands	14 (3)	54 (3)	8 (3)	32 (3)
North East	12 (2)	47 (2)	10 (4)	39 (4)
Northern Ireland	13 (2)	50 (2)	–	–
Scotland	87 (16)	332 (16)	–	–
Wales	75 (14)	276 (13)	–	–
*Age (years)*				
Median (Q1–Q3)	29 (9–43)	29 (9–44)	28 (7–40)	28 (8–40)
0–10	148 (27)	558 (27)	76 (28)	293 (28)
11–17	32 (6)	120 (6)	15 (5)	58 (5)
18–59	305 (56)	1159 (56)	163 (59)	631 (59)
≥ 60	56 (10)	215 (10)	21 (8)	82 (8)
*Follow-up*				
Mean overall follow-up (years, SD)	9.01 (6.89)	11.74 (7.04)	9.51 (7.17)	11.76 (7.07)

*ACH* Achondroplasia; *CPRD* Clinical Practice Research Database; *HES* Hospital Episode Statistics; *N* Total number of individuals; *n* number of individuals in subset; *Q* Quartile; *SD* Standard deviation

### Skeletal and non-skeletal complications (CPRD cohort, primary care only)

Rates of the pre-specified complications, presented by body system and ranked by highest RR, are presented in Table 2. As would be expected for a skeletal disorder, rates of all skeletal complications were statistically significantly higher among ACH cases than controls, with a RR (95% CI) of 3.83 (3.02–4.85). The skeletal complication with the greatest difference in rate between cases and controls was spinal stenosis/cord compression (RR [95% CI] 30.52 [16.28–57.19]). Non-skeletal complications were also reported at significantly higher rates among individuals with ACH than controls, with a RR (95% CI) of 1.80 (1.59–2.03). This was consistent for complications across nearly all non-skeletal body systems, with the highest RRs reported for the developmental (RR [95% CI] 8.84 [4.18–18.72]), neurological (RR [95% CI] 4.67 [3.21–6.80]) and ENT systems (RR [95% CI] 2.98 [2.43–3.65]). Only cardiovascular conditions were similar among the case and control groups (Table 2).

**Table 2:** Rates of skeletal and non-skeletal complications among ACH cases vs controls (CPRD cohort)

Complications by body system category				Specific complications			
Category	n, ER per 100PY*		RR (95% CI)	Specific complication	n, ER per 100PY*		RR (95% CI)
	Cases (N = 541)	Controls (N = 2052)			Cases (N = 541)	Controls (N = 2052)	
All non-skeletal	3612, 73.92	9903, 41.05	1.80 (1.59–2.03)**	–	–	–	–
Developmental	41, 0.84	23, 0.10	8.84 (4.18–18.72)**	Developmental delay	16, 0.33	9, 0.04	8.80 (3.02–25.68)**
				Speech delay	20, 0.41	13, 0.05	7.61 (3.03–19.13)**
				Motor delay	5, 0.10	< 5, NC	NC
Neurological	228, 4.67	241, 1.00	4.67 (3.21–6.80)**	Incontinence/neurogenic bladder or bowel	87, 1.78	78, 0.32	5.54 (3.56–8.63)**
				Paraplegia/parathesia	51, 1.04	55, 0.23	4.58 (2.55–8.22)**
				Seizures/epilepsy	39, 0.80	80, 0.33	2.41 (0.95–6.11)
				Dementia	5, 0.10	24, 0.10	1.02 (0.35–2.98)
				Hydrocephalus/ventriculomegaly	42, 0.86	0, 0.00	Cases only
				Myelopathy	< 5, NC	< 5, NC	NC
				Failure to thrive	< 5, NC	< 5, NC	NC
				Subdural haemorrhage/haematoma	< 5, NC	< 5, NC	NC
Skeletal	459, 9.39	595, 2.47	3.83 (3.02–4.85)	Spinal stenosis/cord compression	138, 2.82	22, 0.09	30.52 (16.28 – 57.19)**
				Spinal deformities	60, 1.23	13, 0.05	22.78 (9.99–51.97)**
				Leg deformity (including genu varum)	15, 0.31	5, 0.02	14.39 (5.45–37.99)**
				Falls	105, 2.15	191, 0.79	2.73 (1.84–4.04)**
				Osteoporosis/osteopenia/reduced BMD	39, 0.80	72, 0.30	2.69 (1.44–5.05)**
				Arthritis/osteoarthritis	95, 1.94	291, 1.21	1.65 (1.17–2.31)**
				Craniocervical stenosis	7, 0.14	0, 0.00	Cases only
				Cauda equina syndrome	< 5, NC	< 5, NC	NC
ENT	517, 10.58	844, 3.50	2.98 (2.43–3.65)**	Hearing loss/deafness	101, 2.07	143, 0.59	3.50 (2.50–4.89)**
				Enlarged tonsils	13, 0.27	18, 0.07	3.34 (1.26–8.86)**
				Otitis media	365, 7.47	565, 2.34	3.11 (2.45–3.94)**
				Voice abnormalities	8, 0.16	16, 0.07	2.47 (0.93–6.57)
				Sinusitis	25, 0.51	101, 0.42	1.24 (0.71–2.16)
				Tracheomalacia/bronchomalacia	< 5, NC	0, 0.00	Cases only
				Middle ear dysfunction/disorder	< 5, NC	< 5, NC	NC
Respiratory***	144, 2.95	250, 1.04	2.84 (1.94–4.17)**	Apnoea/sleep disordered breathing	47, 0.96	9, 0.04	25.81 (10.00–66.60)**
				Sleep disorder	97, 1.99	241, 1.00	1.98 (1.23–3.19)
Metabolic	326, 6.67	984, 4.08	1.65 (1.24–2.18)**	Obesity	213, 4.36	424, 1.76	2.59 (2.26–2.97)**
				Hyperlipidaemia	43, 0.88	157, 0.65	1.36 (0.88–2.10)
				Diabetes	94, 1.92	461, 1.91	1.00 (0.49–2.06)
Mental health	377, 7.72	1156, 4.79	1.62 (1.21–2.17)**	Self-harm/suicidal ideation	9, 0.18	12, 0.05	3.71 (1.17–11.77)**
				ADD/ADHD/adjustment disorder	7, 0.14	10, 0.04	3.44 (1.13–10.51)**
				Depression/anxiety	301, 6.16	991, 4.11	1.51 (1.09–2.08)**
				Substance dependence/abuse	37, 0.76	131, 0.54	1.40 (0.62–3.18)
				‘Other’ mental health issues	23, 0.47	12, 0.05	9.07 (1.99–41.30)**
Cardiovascular	565, 11.56	2359, 9.78	1.17 (0.92–1.49)	Stroke	7, 0.14	23, 0.10	1.56 (0.65–3.74)
				Chest pain/angina	194, 3.97	760, 3.15	1.25 (0.96–1.63)
				Hypertension	321, 6.57	1367, 5.67	1.15 (0.80–1.65)
				Coronary disease	36, 0.74	156, 0.65	1.14 (0.57–2.28)
				Myocardial infarction	7, 0.14	53, 0.22	0.66 (0.28–1.58)
Other	1440, 29.47	4049, 16.78	1.76 (1.52–2.03)**	Pain	1138, 23.29	3047, 12.63	1.84 (1.58–2.15)**
				Gastrointestinal issues	208, 4.26	622, 2.58	1.66 (1.31–2.09)**
				Headache	75, 1.53	264, 1.09	1.41 (0.99–2.03)
				Sexual health/gynaecological issues	19, 0.39	116, 0.48	0.79 (0.38–1.63)

*ACH* Achondroplasia; *ADD* Attention deficit disorder; *ADHD* Attention deficit hyperactivity disorder; *BMD* Bone mineral density; *CI* Confidence interval; *CPRD* Clinical Practice Research Database; *ENT* Ear, nose and throat; *ER* Event rate, *N* Total number of individuals; *n* Number of cases of complication of interest; *NC* Not calculated (due to small cell size); *PY* Person years; *RR* Rate ratio

*Where the number of patients was reported as an integer of more than 0 but less than 5, this has been stated as < 5 due to CPRD reporting requirements

**Statistically significant result (α = 0.05)

***Sleep disorder and apnoea/sleep disordered breathing represent two distinct Read codes; given limited information on what complications would fall under each category, results for these categories have been presented separately

**Fig. 1:**
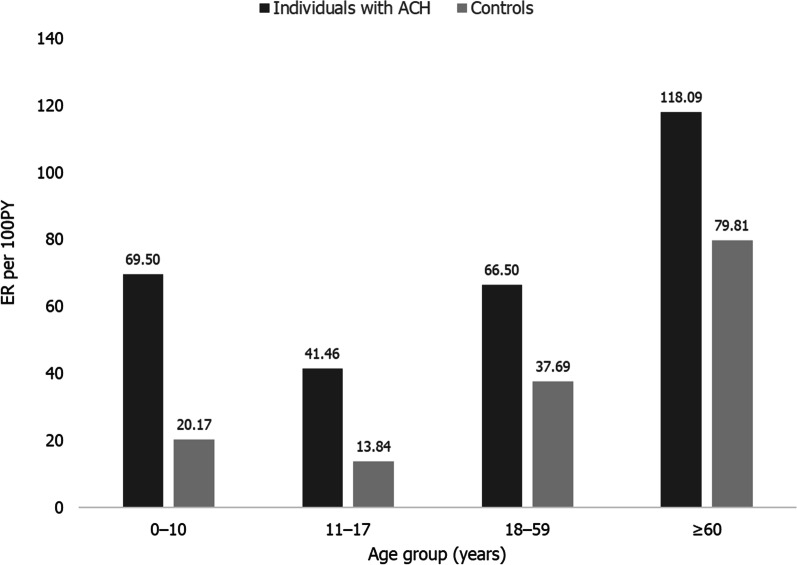
Rates of non-skeletal complications by age among ACH cases vs controls (CPRD cohort). *ACH* Achondroplasia; *CPRD* Clinical Practice Research Database; *ER* Event rate; *PY* Person years

Among ACH cases, the five most commonly reported complications were all non-skeletal and included pain, otitis media, hypertension, depression/anxiety and obesity (Table 2). Rates of skeletal and non-skeletal complications by age group in ACH cases are provided in Table 3. The rates of non-skeletal complications overall were highest among ACH cases between the ages of 0–10 years and above the age of 60 years (Fig. 1). Assessing specific non-skeletal complications, the rates for pain, hypertension, depression/anxiety, incontinence/neurogenic bladder or bowel, sleep disorder and obesity increased with age; rates of otitis media and apnoea/sleep disordered breathing decreased after the age of 18 years (Table 3).

**Table 3:** Rates of skeletal and non-skeletal complications by age group among ACH cases (CPRD cohort)

Complications by body system category and age group					Specific events by age group*		
Category	n, ER per 100PY**				Specific event	n, ER per 100PY**	
	0–10 years	11–17 years	18–59 years	≥ 60 years		< 18 years	≥ 18 years
All non-skeletal	465, 69.50	169, 41.46	1960, 66.50	1018, 118.09	–	–	–
Developmental	29, 4.33	< 5, NC	10, 0.34	< 5, NC	Developmental delay	13, 1.21	< 5, NC
					Speech delay	12, 1.11	8, 0.21
					Motor delay	5, 0.46	< 5, NC
Neurological***	37, 5.53	15, 3.68	116, 3.94	60, 6.96	Incontinence/neurogenic bladder or bowel	17, 1.58	70, 1.84
					Paraplegia/parathesia	< 5, NC	51, 1.34
					Seizures/epilepsy	< 5, NC	37, 0.97
					Hydrocephalus/ventriculomegaly	31, 2.88	11, 0.29
					Dementia	< 5, NC	5, 0.13
Skeletal***	66, 9.86	23, 5.64	227, 7.70	143, 16.59	Spinal stenosis/cord compression	14, 1.30	124, 3.26
					Arthritis/osteoarthritis	< 5, NC	< 100, 2.47
					Falls	15, 1.39	90, 2.36
					Spinal deformities	46, 4.27	14, 0.37
					Osteoporosis/osteopenia/reduced BMD	0	39, 1.02
					Leg deformity (including genu varum)	< 15, 1.02	< 5, NC
					Craniocervical stenosis	< 5, NC	< 10, 0.13
ENT***	271, 40.51	42, 10.30	167, 5.67	37, 4.29	Otitis media	254, 23.59	111, 2.91
					Hearing loss/deafness	45, 4.18	56, 1.47
					Sinusitis	0	25, 0.66
					Enlarged tonsils	< 15, 0.93	< 5, 0.08
					Voice abnormalities	0	8, 0.21
Respiratory	35, 5.23	< 5, NC	73, 2.48	< 35, NC	Sleep disorder	6, 0.56	91, 2.39
					Apnoea/sleep disordered breathing	33, 3.07	14, 0.37
Metabolic	< 5, NC	< 10, NC	213, 7.23	103, 11.95	Obesity	10, 0.93	179, 4.70
					Diabetes	< 5, NC	94, 2.47
					Hyperlipidaemia	< 5, NC	43, 1.13
Mental health	< 5, NC	13, 3.19	254, 8.62	107, 12.41	Depression/anxiety	7, 0.65	294, 7.72
					Substance dependence/abuse	< 5, NC	37, 0.97
					‘Other’ mental health issues	< 5, NC	22, 0.58
					Self-harm/suicidal ideation	5, 0.46	< 5, NC
					ADD/ADHD/adjustment disorder	< 5, NC	< 5, NC
Cardiovascular	< 10, NC	< 10, NC	282, 9.57	271, 31.44	Hypertension	< 5, NC	319, 8.37
					Chest pain/angina	10, 0.93	184, 4.83
					Coronary disease	< 5, NC	36, 0.95
					Stroke	< 5, NC	7, 0.18
					Myocardial infarction	< 5, NC	7, 0.18
Other	84, 12.56	83, 20.36	861, 29.21	412, 47.79	Pain	96, 8.92	1042, 27.35
					Gastrointestinal issues	43, 3.99	165, 4.33
					Headache	28, 2.60	47, 1.23
					Sexual health/gynaecological issues	< 5, NC	19, 0.50

*ACH* Achondroplasia; *ADD* Attention deficit disorder; *ADHD* Attention deficit hyperactivity disorder; *BMD* Bone mineral density; *CPRD* Clinical Practice Research Database; *ENT* Ear, nose and throat; *ER* Event rate; *n* Number of events of interest; *NC* Not calculated (due to small cell size); *PY* Person years

*Due to small cell sizes, specific events by age group are presented within broader < 18 or ≥ 18 year age categories

**Where the number of events in a cell was less than 5, this has been stated as < 5 due to CPRD reporting requirements. In some cases, values greater than 5 have been reported as less than (< the next highest multiple of 5) to prevent calculation of exact values elsewhere

***Data on myelopathy, failure to thrive, subdural haemorrhage/haematoma, cauda equinasyndrome, tracheomalacia/bronchomalacia and middle ear dysfunction/disorder have not been reported by age group, since the overall number of events was < 5

### Medication use (CPRD cohort, primary care only)

Individuals with ACH consistently showed significantly higher rates of medication use in primary care compared to controls (RR [95% CI] 1.69 [1.42–2.02]), with the exception of metabolism/endocrine and cardiovascular medications (Table 4).

**Table 4:** Rates of medication use among ACH cases vs controls (CPRD cohort)

Medications by category				Specific medications			
Category	n, ER per 100PY*		RR (95% CI)	Type	n, ER per 100PY*		RR (95% CI)
	Cases (N = 541)	Controls (N = 2052)			Cases (N = 541)	Controls (N = 2052)	
Any prescription	49,070, 1004.27	146,315, 606.49	1.69 (1.42–2.02)**	–	–	–	–
Genito-urinary^†^	1731, 35.43	1499, 6.21	5.75 (2.85–11.58)**	Urinary incontinence	1731, 35.43	1499, 6.21	5.75 (2.85–11.58)**
Musculoskeletal	15,852, 324.43	30,899, 128.08	2.57 (2.00–3.31)**	Pain	13,543, 277.17	25,952, 107.57	2.61 (2.03–3.35)**
				Bone disorders	2309, 47.23	4947, 20.51	2.32 (1.40–3.85)**
Nervous system	8554, 175.1	20,790, 86.2	2.09 (1.57–2.77)**	Dementia	156, 3.19	256, 1.06	3.15 (0.82–12.15)
				Anti-epileptics	1677, 34.32	2767, 11.47	2.99 (1.45–6.15)**
				Antidepressants/anxiolytics	6013, 123.06	15,177, 62.91	2.02 (1.52–2.68)**
				ADHD	93, 1.90	309, 1.28	1.48 (0.21–10.62)
				Substance dependence	75, 1.53	296, 1.23	1.27 (0.60–2.68)
Antibiotics/Antifungals^‡^	4478, 91.65	12,951, 53.68	1.71 (1.43–2.03)**	Antibiotics/antifungals**	4478, 91.65	12,951, 53.68	1.71 (1.43–2.03)**
Digestive system	4840, 99.06	14,292, 59.24	1.70 (1.28–2.24)**	Proton pump inhibitors	4151, 84.95	11,632, 48.22	1.80 (1.36–2.37)**
				Gastrointestinal	689, 14.10	2660, 11.03	1.28 (0.65–2.51)
Metabolism and endocrine	1803, 36.90	7156, 29.66	1.24 (0.64–2.40)	Anti-obesity	97, 1.99	248, 1.03	1.93 (0.82–4.54)
				Diabetes treatments	1706, 34.91	6908, 28.63	1.22 (0.61–2.43)
Cardiovascular	11,384, 232.98	56,929, 235.98	1.01 (0.78–1.30)	Anti-hypertensives	5,738, 117.43	24,553, 101.77	1.18 (0.89–1.56)
				Cardiac medications	2955, 60.48	14,854, 61.57	0.99 (0.66–1.50)
				Statins	2691, 55.07	17,522, 72.63	0.77 (0.54–1.08)

*ACH* Achondroplasia; *ADHD* Attention deficit hyperactivity disorder; *CI* Confidence interval; *CPRD* Clinical Practice Research Database; *ER* Event rate; *N* Total number of individuals; *n* Number of uses of medications of interest; *PY* Person years; *RR* Rate ratio

*Where the number of patients was reported as an integer of more than 0 but less than 5, this has been stated as < 5 due to CPRD reporting requirements

**Statistically significant result (α = 0.05)

^**†**^Genito-urinary system-related medications only included medication for urinary incontinence

^**‡**^Antibiotic/Antifungal medications were not broken down into specific medication types

Among ACH cases, the most commonly prescribed medications (by ER per 100PY) were pain medications, antidepressants/anxiolytics and antibiotics/antifungals (Table 4). Rates by age are provided in Additional file 4. The use of pain medications, antidepressants/anxiolytics, proton pump inhibitors and medications for urinary incontinence and diabetes increased in individuals above the age of 18 years (Additional file 4). The use of anti-hypertensives, anti-obesity medications, cardiac medications, statins and medications for bone disorders, dementia and substance dependence were only reported in individuals above the age of 18 years. The only medication type occurring at a higher rate among individuals less than 18 years old was attention deficit hyperactivity disorder (ADHD) medication (Additional file 4).

### GP referrals to specialist care (CPRD cohort, primary care only)

GPs made referrals to a wide range of specialists among both cases and controls, but ACH cases were referred by GPs to specialist care almost twice as frequently as controls (RR [95% CI] 1.93 [1.66–2.24]; Additional file 4). The highest RRs were recorded for referrals for wheelchair use and occupational therapy, though the actual proportions of ACH cases that received these referrals were small (0.92% and 2.40%, respectively).

Individuals with ACH were most often referred to general medicine, orthopaedics, specialist imaging, physiotherapy and ear, nose and throat (ENT) specialists (Additional file 5).

When analysed by age, rates of GP referrals were greatest above the age of 18 years. Across all ACH age groups, referrals to general medicine were the most common. Individuals less than 18 years old were also frequently referred to paediatrics, ENT and physiotherapy, while those above the age of 18 years were frequently referred to orthopaedics, physiotherapy and specialist imaging.

### Surgical procedures (CPRD HES-linked cohort)

The rate of any surgical procedure of interest were almost ten times greater among ACH cases than controls (RR [95% CI] 9.83 [7.09–13.61]; Table 5). As would be expected for a skeletal condition, the rate of orthopaedic surgeries was significantly higher among ACH cases, and several orthopaedic/spinal procedures including foramen magnum decompression and limb lengthening reported only among the case population. However, rates of procedures related to ENT issues were also significantly greater among cases than controls, with grommet insertions around 13 times more likely to occur in individuals with ACH. Surgeries on the respiratory and cardiovascular systems (such as ventilation procedures and cardiac stent insertions) were uncommon in both cases and controls.

**Table 5:** Hospital surgical procedure rates among ACH cases vs controls (CPRD HES-linked cohort)

Procedures by body system				Specific procedures			
Body system	n, ER per 100PY*		RR (95% CI)	Type	n, ER per 100PY*		RR (95% CI)
	Cases (N = 275)	Controls (N = 1064)			Cases (N = 275)	Controls (N = 1064)	
Any procedure	265, 5.15	108, 0.53	9.83 (7.09–13.61)**	–	–	–	–
Orthopaedic	141, 2.74	53, 0.26	10.63 (6.97–16.22)**	–	–	–	–
Spinal	76, 1.48	21, 0.10	14.46 (8.73–23.94)**	Spinal decompression	69, 1.34	21, 0.10	13.13 (7.87–21.89)**
				Foramen magnum decompression	7, 0.14	0, 0.0	Cases only
Limb	65, 1.26	32, 0.16	8.10 (4.22–15.56)**	Bone fixation†	44, 0.86	11, 0.05	15.99 (7.16–35.75)**
				Hip/knee replacement	10, 0.19	21, 0.10	1.90 (0.64–5.67)
				Limb lengthening	10, 0.19	0, 0.0	Cases only
				Brace provision	< 5, NC	0, 0.0	Cases only
ENT	104, 2.02	48, 0.23	8.62 (5.01–14.82)**	Grommet insertion	66, 1.28	20, 0.10	13.15 (5.55–31.17)**
				Tonsillectomy/adenoidectomy	23, 0.45	23, 0.11	3.99 (2.25–7.07)**
				Hearing Aid	6, 0.12	< 5, NC	NC
				Tympanoplasty	9, 0.18	< 5, NC	NC
Neurological	7, 0.12	0, 0.0	Cases only	Shunt/ventriculostomy	7, 0.14	0, 0.0	Cases only
Respiratory	11.0, 0.21	< 5, NC	NC	Tracheostomy	< 5, NC	0, 0.0	Cases only
				Ventilation	10, 0.19	< 5, NC	NC
Cardiovascular	< 5, NC	< 5, NC	NC	Cardiac stent	< 5, NC	< 5, NC	NC

*ACH* Achondroplasia; *CI* Confidence interval; *CPRD* Clinical Practice Research Database; *ENT* Ear, nose and throat; *ER* Event rate; *HES* Hospital Episode Statistics; *N* Total number of individuals; *n* Number of occurrences of procedure of interest; *NC* Not calculated (due to small cell size); *PY* Person years; *RR* Rate ratio

*Where the number of patients was reported as an integer of more than 0 but less than 5, this has been stated as < 5 due to CPRD reporting requirements

**Statistically significant result (α = 0.05)

^†^Bone fixation procedures were any procedures that involved internal or external bone fixation; for example, fixation related to fracture stabilisation. Bone fixation for limb lengthening was coded under ‘limb lengthening’; however, fractures caused by or associated with limb-lengthening may have been coded under ‘bone fixation’

Among individuals with ACH, the three most common types of surgical procedures included two orthopaedic procedures (spinal decompression surgery and bone fixation procedures for any cause) and one non-skeletal procedure (grommet insertion; Table 5). Within the case population, the rate of any surgical procedure was highest among individuals aged 0–10 (17 per 100PY) and steadily decreased among older age groups (Additional file 4). This pattern was consistent across all body systems, with the exception of orthopaedic, for which the highest rate was among 11–17 year-olds (Additional file 4).

### Healthcare visits (CPRD HES-linked cohort)

The mean annual number of GP consultations, hospital outpatient visits and hospital in-patient admissions per person was significantly higher among ACH cases than controls (Table 6). In addition, the mean (SD) length of inpatient stay (in days) was higher among individuals with ACH than controls (cases: 4.13 [13.16]; controls: 2.30 [6.54]).

**Table 6:** Healthcare visits among ACH cases vs controls, overall and by age (CPRD HES-linked cohort)

Healthcare visit type	Age group (years)	Mean number of visits per person per year (SD)		*p*-value	Mean length of inpatient stay, days (SD)	
		Cases (N = 275)	Controls (N = 1052)		Cases (N = 275)	Controls (N = 1052)
GP Consultations	Overall	8.02 (7.99)	5.10 (4.96)	*p* < 0.0001*	N/A	
	0–10	5.35 (6.98)	3.47 (3.49)	*p* < 0.005*		
	11–17	12.83 (6.61)	1.00 (1.54)	*p* < 0.005*		
	18–59	6.60 (7.02)	3.96 (4.18)	*p* < 0.0001*		
	≥ 60	8.23 (7.84)	6.37 (6.29)	*p* < 0.005*		
Hospital outpatient visits	Overall	3.02 (3.56)	1.37 (1.87)	*p* < 0.0001*	N/A	
	0–10	3.52 (3.59)	0.83 (1.40)	*p* < 0.0001*		
	11–17	1.71 (1.65)	0.40 (0.73)	*p* < 0.0001*		
	18–59	1.67 (1.83)	1.07 (1.42)	*p* < 0.0001*		
	≥ 60	2.53 (5.25)	1.83 (2.29)	*p* = 0.1464		
Hospital in-patient admissions	Overall	0.51 (0.77)	0.26 (0.35)	*p* < 0.0001*	4.13 (13.16)	2.30 (6.54)
	0–10	0.72 (1.17)	0.23 (0.40)	*p* < 0.0001*	1.68 (6.03)	1.40 (3.57)
	11–17	0.18 (0.16)	0.10 (0.17)	*p* < 0.005*	3.28 (10.98)	1.65 (6.25)
	18–59	0.25 (0.25)	0.19 (0.21)	*p* < 0.005*	5.01 (16.02)	1.63 (4.56)
	≥ 60	0.63 (0.78)	0.36 (0.43)	*p* < 0.005*	6.45 (14.73)	3.91 (9.49)

*ACH* Achondroplasia; *CPRD* Clinical Practice Research Database; *ER* Event rate; *GP* General practitioner; *N/A* Not applicable; *PY* Person years; *RR* Rate ratio; *SD* Standard deviation

*Statistically significant result (α = 0.05)

Among the ACH case population, the mean annual number of visits per person was greatest for GP consultations, followed by outpatient visits and in-patient admissions (Table 6). When analysed by age among individuals with ACH, the mean annual number of GP consultations generally increased with age. The mean annual number of outpatient visits and in-patient admissions was greatest in the youngest (0–10 years) and oldest (greater than 60 years) age groups. The mean (SD) length of in-patient stay increased with age, from 1.68 (6.03) days for 0–10 years to 6.45 (14.73) days for those greater than 60 years.

### Medical imaging (CPRD HES-linked cohort)

The overall rate of medical imaging procedures was more than twice as high for ACH cases than controls (RR [95% CI] 2.48 [1.97–3.11]). By specific procedure types, RRs (95% CI) demonstrate that rates were consistently significantly higher among individuals with ACH than matched controls (neuroimaging: 7.02 [2.69–18.36]; skeletal imaging: 2.50 [1.99–3.15]; spinal imaging: 7.50 [4.92–11.43] and bone density according to dual-energy X-ray absorptiometry [DEXA] scans: 2.86 [1.07–7.63]), with the exception of cardiac imaging where there was no significant difference (RR [95% CI] 1.04 [0.57–1.90]).

Among ACH cases, rates were greatest for skeletal imaging, followed by spinal imaging. By age, rates for any imaging procedure were highest in individuals with ACH aged 0–10, driven primarily by the high rate of skeletal imaging procedures in this age group compared to other ages.

### Mortality (CPRD HES-linked cohort)

Table 7 presents mortality by gender and age group among cases and controls. Overall, the mortality rate within the follow-up period was almost twice as high among ACH cases than controls (RR [95% CI] 1.90 [1.18–3.06]). Within the control group, 81% of deaths were among individuals greater than 60 years old, while for patients with ACH, deaths were similarly distributed between the 18–59 and the greater than 60 years age groups. Deaths in female individuals represented a slightly higher proportion than males in both case and control groups (61% and 59%, respectively). The underlying cause of death (by ICD-10 chapter) was assessed and is included in Additional file 6. However, reporting of this information is limited due to data disclosure requirements whereby cell values of more than 0 but less than 5 must be masked and stated as < 5. Among both cases and controls, the most common causes of death were diseases of the circulatory system and neoplasms; mortality rates for these causes were not significantly different between cases and controls (RR [95% CI] 1.2 [0.5–2.9] and RR [95% CI] 1.1 [0.4–2.8] respectively). The next highest cause of death among cases was congenital malformations, deformations and chromosomal abnormalities (5/31 [16.1%] of deaths), while diseases of the digestive system and respiratory systems represented the next highest causes of death among controls (8/69 [11.6%] and 7/69 [10.1%], respectively) (Additional file 6).

**Table 7:** Mortality by gender and age among ACH cases versus controls (CPRD HES-linked cohort)

	Cases (N = 275)	Controls (N = 1052)
*All reported deaths*		
Number of deaths, n	31	36
ER per 100PY	0.60	0.34
RR (95% CI)	1.90 (1.18–3.06)	
*Gender, n (% of total deaths)*		
Male	12 (39)	28 (41)
Female	19 (61)	41 (59)
*Age, n (% of total deaths)*		
0–10	0	0
11–17	0	0
18–59	14 (45)	13 (19)
60 +	17 (55)	56 (81)

*ACH* Achondroplasia; *CI* Confidence interval; *CPRD* Clinical Practice Research Database; *ER* Event rate; *HES* Hospital Episode Statistics; *N* Total number of individuals; *n* Number of individuals in subset; *RR* Rate ratio

## Discussion

This retrospective, observational cohort study utilised rich sources of routinely collected health data in the UK to gain a more comprehensive understanding of non-skeletal complications, surgical procedures, healthcare needs and mortality of individuals with ACH. Importantly, the study included a large sample of individuals with ACH from across the age spectrum, including substantial numbers of adults, who have been studied relatively less [21]. Furthermore, the study included a matched control group from within the same population and utilised both primary and secondary care data sources, providing an enhanced assessment of outcomes. It also had an extended follow-up period of around 10 years.

As ACH is a disorder of endochondral bone growth, rates of skeletal complications were expected to be elevated among individuals with ACH compared to matched controls. In line with this, the rate of any skeletal complication in this study population was more than four times higher among individuals with ACH than controls, providing an internal control of the methodology. However, ACH cases also had higher rates of non-skeletal complications, with patients in the youngest and oldest age groups experiencing the greatest burden. Some of these non-skeletal complications may be mechanistically attributed to abnormal bone formation (for instance, spinal stenosis). Others may be secondary or downstream effects of skeletal complications; pain, for example, can arise as a secondary impact of skeletal complications but could itself be contributing to reduced quality of life and the occurrence of serious mental health conditions. Others, such as obesity and gastrointestinal issues, are less clearly related to skeletal effects and the underlying cause of these complications in ACH remains unknown. Their occurrence may be related to upregulated *FGFR3* signalling in non-skeletal tissues; mRNA and protein studies have shown that *FGFR3* expression takes place across several tissues, particularly in the brain, gut and skin [23–25]. The present study builds upon the existing literature to demonstrate the significant burden that non-skeletal complications can have on the health of people living with ACH, and suggests a need for more detailed studies to understand the causes of these manifestations. In defining their cause and effect more clearly, it could then be established if non-skeletal complications are modifiable with new treatments.

The findings from this study suggest a significantly higher rate of developmental delay and speech delay among individuals with ACH versus controls. Recently published guidance for achondroplasia recommends that the development of individuals with ACH is monitored against normative development charts for individuals with ACH [26]. Whilst differences between the development of individuals with ACH versus the general population have been well-described [4, 26], rates of developmental or speech delay within ACH specifically have not been reported. This is therefore an area requiring further investigation, so that healthcare professionals can be aware of potential delays to developmental milestones within ACH [26]. Similarly, the low proportion (2.40%) of ACH cases referred to occupational therapists by GPs represents opportunity to review and improve occupational therapy referral practices for individuals with ACH. The study also highlighted that individuals with ACH more frequently experience pain, urinary incontinence, problems with the digestive system, seizures and depression/anxiety than the general UK population, require increased medication use and have an increased surgical burden across age groups. These findings support and add to the existing literature base [5, 13, 14, 21, 27], while highlighting the multi-disciplinary care needed for those with ACH [26].

In contrast to all other body systems, the cardiovascular system consistently did not show significant differences between cases and controls for complications, medication use, medical imaging and mortality. This is particularly interesting given the significantly higher rates of obesity, a known risk factor for cardiovascular problems [28], seen within the ACH case population. Obesity is also a risk factor for diabetes and hyperlipidaemia, and the rates of these complications were also found to be similar between ACH cases and controls. These findings are supportive of other recent studies which indicate that, despite individuals with ACH having a high body mass index (BMI), diabetes risk [29] and cardiovascular outcomes are low [20, 30]. The lack of significant differences may be due to some less known pathogenic mechanism related to *FGFR3* dysregulation itself, but this remains to be elucidated.

Finally, the study offers insights into the rates and causes of mortality. Among this population sample, of which almost two thirds were adults, we report increased mortality of almost two-fold among adults with ACH compared to controls, notably among the 18–59 year-old age group. The doubling of risk is in keeping with previous studies reporting standardised mortality ratios of 2.27 (95% CI 1.7–3.0) [15] and 2.05 (95% CI 1.75–2.40) [16] in ACH populations from the US. Previous studies have demonstrated an increased mortality among children [17]. However, our study was likely underpowered to detect deaths in this age group. While a small sample size also hampered full exploration of cause of death, the top two causes of death (circulatory conditions and cancers) were the same in both ACH individuals and the general population within this study.

This study had several limitations. Despite a large overall sample size for a rare disease, there was low statistical power among individual outcome categories, especially among complications, surgeries and causes of death, where sample sizes were small. Furthermore, terms of use for the CPRD database did not permit full disclosure of information on outcomes where sample size was less than five. The use of pre-specified categories of interest for a number of outcomes may have restricted the capacity to capture unexpected or novel findings. Additionally, whilst the authors have separately discussed skeletal and non-skeletal complications, it is acknowledged that the distinction between these categories is not clear cut, as non-skeletal complications may be secondary to skeletal issues. Finally, it is possible that these results, which are specific to the UK databases utilised, may lack generalisability to ACH cases being treated in other countries with differing standards of care; this is particularly relevant to the healthcare needs outcomes.

## Conclusions

As the first study which uses routinely collected data to compare the rates of complications, healthcare needs and mortality between UK ACH and control populations, the findings presented here offer important insights into the experiences of individuals with ACH. These data demonstrate that individuals with ACH face significantly higher rates of complications, not only with respect to skeletal manifestations, but also across non-skeletal body systems when compared to matched controls. The study findings also indicate that individuals with ACH have greater healthcare needs compared to the general population, emphasising the need for an optimised approach to patient care. For example, the development of specialised services and a greater focus on preventing complications could better support ACH patients in the UK and optimise their need for regular healthcare visits and surgical procedures.

## Supplementary Information

**Additional file 1.** Additional details on datasets and linkage.

**Additional file 2.** Methodology for identifying cases, and matching to controls.

**Additional file 3.** Dwarfism causing and growth-affecting conditions used as exclusionary codes to define study controls.

**Additional file 4.** Outcomes by age group among ACH cases (CPRD and CPRD HES-linked cohorts).

**Additional file 5.** Rate of GP referrals to specialist care among cases and controls (CPRD cohort).

**Additional file 6.** Underlying cause of death by ICD-10 chapter among ACH cases vs controls (CPRD HES-linked cohort).

## Data Availability

Access to CPRD data used for the study is not publicly available but can be accessed subject to protocol approval via CPRD’s Research Data Governance (RDG) Process and license fees. Instructions on submitting an application to access the CPRD data can be found http://www.cprd.com/data-access.
