# Supplementary material for: Higher rates of non-skeletal complications and greater healthcare needs in achondroplasia compared to the general UK population: a matched cohort study using the CPRD database
Source: Orphanet J Rare Dis. 2023 Jul 25;18:211. doi: 10.1186/s13023-023-02811-5 (PMC10367327; doi:10.1186/s13023-023-02811-5)
Supplement: Supplementary file 1 — Additional file 1. Additional details on datasets and linkage. [file 13023_2023_2811_MOESM1_ESM.docx]

Additional File 1: Additional details on datasets and linkage

*Overview of included datasets*

Data were available from the included datasets over the following date ranges:

| **Data source** | **Start** | **End** |
| --- | --- | --- |
| CPRD | 01-Jan-1987 | 31-Dec-2018 |
| HES APC | 04-Jan-1997 | 31-Dec-2017 |
| HES OP | 04-Jan-2003 | 31-Dec-2017 |
| HES DID | 04-Jan-2012 | 31-Oct-2017 |
| ONS DEATH | 01-Feb-1998 | 13-Feb-2018 |

Linked HES data were available for 2,150 eligible patients in addition to the CPRD GOLD primary care data.

**Abbreviations:** APC, Admitted Patient Care; CPRD, Clinical Practice Research Database; DID, Diagnostic Imaging Dataset; HES, Hospital Episode Statistics; ONS, Office of National Statistics; OP, Outpatient Data.

*CPRD definitions of ‘acceptable’ patients*

Patients are labelled as ‘acceptable’ for use in research by a process that identifies and excludes patients with non-continuous follow up or patients with poor data recording that raises suspicion as to the validity of the that patients record.
