# Supplementary material for: Higher rates of non-skeletal complications and greater healthcare needs in achondroplasia compared to the general UK population: a matched cohort study using the CPRD database
Source: Orphanet J Rare Dis. 2023 Jul 25;18:211. doi: 10.1186/s13023-023-02811-5 (PMC10367327; doi:10.1186/s13023-023-02811-5)
Supplement: Supplementary file 2 — Additional file 2. Methodology for identifying cases, and matching to controls. [file 13023_2023_2811_MOESM2_ESM.docx]

Additional File 2: Methodology for identifying cases, and matching to controls

1) Identification of a broad cohort

A broad cohort of possible ACH cases was defined using specific codes for the CPRD database, and a non-specific code for the HES database, as outlined in the table below.

| **Condition** | **Codes** | |
| --- | --- | --- |
| **CPRD Database** | **Medcode** | **Read code** |
| Achondroplasia | 12198 | PG41.00 |
| Hypochondroplasia | 94173/29840 | PG41000/PG42.15 |
| Pseudoachondroplasia | 43115/36351 | PG44600/PG42.17 |
| **HES Database** | **ICD-10** | |
| Pseudoachondroplasia | Q77.4 | |

Abbreviations: CPRD, Clinical Practice Research Database; HES, Hospital Episode Statistics; ICD-10: International Classification of Diseases 10^th^ Revision.

2) Identification of definitive ACH cases via CPRD codes

The broad cohort was then filtered to identify definitive ACH cases. Definitive cases were defined as records:

- Which only contained specific codes for ACH in CPRD (i.e. excluding records which had a specific code of hypochondroplasia or pseudoachondroplasia)
- Which had a non-specific ICD-10 code in HES, in addition to only specific codes for ACH in CPRD (i.e. excluding records which had a second code from CPRD of hypochondroplasia or pseudoachondroplasia)

| **Record in CPRD only** | | |
| --- | --- | --- |
| Single code | *Achondroplasia* | *Include* |
|  | Hypochondroplasia | Exclude |
|  | Pseudoachondroplasia | Exclude |
| Multiple codes | Achondroplasia followed by hypochondroplasia/pseudoachondroplasia; hypochondroplasia followed by pseudoachondroplasia; pseudoachondroplasia followed by achondroplasia | Exclude |

| **Record in HES and CPRD** | | |
| --- | --- | --- |
|  | *Q77.4 + achondroplasia* | *Include* |
|  | Q77.4 + hypochondroplasia/pseudoachondroplasia | Exclude |

Footnote: Definitive cases in italics.

Abbreviations: CPRD, Clinical Practice Research Database; HES, Hospital Episode Statistics.

3) Review of HES cases for further definitive cases

There were additional potential cases which only had a HES code (i.e. which had no CPRD code). These cases were further investigated by assessing height records or date at first ICD-10 code, in order to distinguish between achondroplasia and hypochondroplasia cases.

Based on input from clinical authors, ACH cases are likely to be diagnosed early after birth, whereas there is higher likelihood of hypochondroplasia cases being given a diagnosis later (>2 years) as clinical presentation becomes more apparent. Therefore potential HES cases were divided into 4 groups as follows:

| 1 | Age at first code ≥ 2 years and no height record in dataset | Exclude |
| --- | --- | --- |
| *2* | *Age at first code ≤ 2 years and no height record in dataset* | *Include* |
| 3 | Height records present after age 18 years (adult height) | Based on height data* |
| 4 | Height records present before 18 years |  |

*Inclusion criteria for groups 3 and 4 were based on available height data for the patients, with this height data compared to achondroplasia-specific height reference data from Hoover-Fong (2021).^[[1]](#footnote-1)^

Cases with measurements within the range of the 5^th^ to 95^th^ percentiles (by gender) were included within the dataset. Data were read off charts using WebPlot Digitizer (Automeris). For adults with multiple height measurements, an average of measurements was used, whereas for children, the tallest height measurement was used.

4) Cohort matching

Up to 4 controls were matched to each ACH individual based on GP practice, year of birth (+/- 1 year), sex and linkage eligibility to HES using index date matching, with the index date for ACH cases defined as the first record of ACH in the CPRD or HES APC database during the study period (as illustrated in the diagram below). The matching index date of an individual with ACH was required to fall between the follow-up start and follow-up end dates of the relevant control(s).

**Control Patient** Follow-up Start Follow-up End

Date

**Case Patient** Matching Index date

The matching index date was the same as the index date if the index date fell after the patient registration date. If the index date was prior to the patient registration date, the matching index date was defined as the latest of the registration date and follow-up start date.

For control patients, the index date was taken as the matching index date.

1. Hoover-Fong JE, Schulze KJ, Alade AY, Bober MB, Gough E, Hashmi SS, Hecht JT, Legare JM, Little ME, Modaff P, Pauli RM. Growth in achondroplasia including stature, weight, weight-for-height and head circumference from CLARITY: achondroplasia natural history study—a multi-center retrospective cohort study of achondroplasia in the US. Orphanet journal of rare diseases. 2021 Dec;16(1):1-9. [↑](#footnote-ref-1)
