# Supplementary material for: Higher rates of non-skeletal complications and greater healthcare needs in achondroplasia compared to the general UK population: a matched cohort study using the CPRD database
Source: Orphanet J Rare Dis. 2023 Jul 25;18:211. doi: 10.1186/s13023-023-02811-5 (PMC10367327; doi:10.1186/s13023-023-02811-5)
Supplement: Supplementary file 3 — Additional file 3. Dwarfism causing and growth-affecting conditions used as exclusionary codes to define study controls. [file 13023_2023_2811_MOESM3_ESM.docx]

**Additional File 3: Dwarfism causing and growth-affecting conditions used as exclusionary codes to define study controls**

General population controls were defined as those without growth disorders including short stature conditions, using the codes below.

| **Condition** | **Medcode** | **Read code** |
| --- | --- | --- |
| [M]Chondroblastic osteosarcoma | 24539 | BBV2.00 |
| [M]Chondroblastoma NOS | 68524 | BBW7.00 |
| [M]Chondroblastoma, malignant | 98559 | BBW8.00 |
| [M]Chondroma NOS | 50796 | BBW2.00 |
| [M]Chondromatosis NOS | 33589 | BBW3.00 |
| [M]Chondromatous neoplasm NOS | 33973 | BBWz.00 |
| [M]Chondromatous neoplasms | 36503 | BBW..00 |
| [M]Chondromyxoid fibroma | 49568 | BBWA.00 |
| [M]Chondrosarcoma NOS | 7941 | BBW4.00 |
| [M]Juxtacortical chondroma | 49667 | BBW5.00 |
| [M]Juxtacortical chondrosarcoma | 63659 | BBW6.00 |
| [M]Mesenchymal chondrosarcoma | 52684 | BBW9.00 |
| [M]Myxoid chondrosarcoma | 4118 | BBV9.00 |
| [M]Osteochondroma | 3166 | BBW0.00 |
| [M]Osteochondromatosis NOS | 16204 | BBW1.00 |
| [X]Chondropathies | 97433 | NyuD.00 |
| [X]Other chondrocalcinosis | 97460 | Nyu1800 |
| [X]Other sp cified hypothyroidism | 73107 | Cyu1100 |
| [X]Other specified juvenile osteochondrosis | 108528 | NyuD200 |
| [X]Other specified osteochondropathies | 55217 | NyuD300 |
| [X]Other variants of Turner's syndrome | 109386 | PyuA500 |
| [X]Spinal osteochondrosis, unspecified | 100102 | Nyu5B00 |
| Aarskog syndrome | 22048 | PKy6500 |
| Achondrogenesis | 43119 | PG4B000 |
| Achondroplasia | - | PG41 |
| Acquired atrophy of thyroid | 46345 | C045.00 |
| Acquired hypothyroidism | 3290 | C04..00 |
| Acromesomolic dysplasia | 89328 | PG44400 |
| Active rickets | 31367 | C280.00 |
| Adult osteochondrosis of spine | 36510 | N32y000 |
| Amsterdam dwarf | 50457 | PKy6000 |
| Apophysitis NOS | 7062 | N32z000 |
| Asphyxiating thoracic dysplasia | 65935 | PG43.00 |
| Blount's disease - osteochondrosis of proximal tibia | 34920 | N324200 |
| Camptomelia dysplasia | 68768 | PG4B400 |
| Chondrocalcinosis | 5701 | N02..11 |
| Chondrocalcinosis due to dicalcium phosphate crystals | 66167 | N020.00 |
| Chondrocalcinosis due to dicalcium phosphate crystals, NOS | 106257 | N020z00 |
| Chondrocalcinosis due to pyrophosphate crystals | 36782 | N021.00 |
| Chondrocalcinosis due to pyrophosphate crystals, NOS | 73696 | N021z00 |
| Chondrocalcinosis NOS | 60833 | N022z00 |
| Chondrocalcinosis unspecified, of multiple sites | 110541 | N022x00 |
| Chondrocalcinosis unspecified, of other specified site | 64371 | N022y00 |
| Chondrocalcinosis unspecified, of the ankle and foot | 98943 | N022700 |
| Chondrocalcinosis unspecified, of the hand | 112191 | N022400 |
| Chondrocalcinosis unspecified, of the lower leg | 69245 | N022600 |
| Chondrocalcinosis unspecified, of the pelvic region/thigh | 73642 | N022500 |
| Chondrocalcinosis unspecified, of the shoulder region | 104671 | N022100 |
| Chondrocalcinosis unspecified, of unspecified site | 69872 | N022000 |
| Chondrocalcinosis, unspecified | 51244 | N022.00 |
| Chondrocalcinosis-dicalcium phosphate, of multiple sites | 98756 | N020x00 |
| Chondrocalcinosis-dicalcium phosphate, of the ankle and foot | 63753 | N020700 |
| Chondrocalcinosis-dicalcium phosphate, of the forearm | 102721 | N020300 |
| Chondrocalcinosis-dicalcium phosphate, of the hand | 94537 | N020400 |
| Chondrocalcinosis-dicalcium phosphate, of the lower leg | 69242 | N020600 |
| Chondrocalcinosis-pyrophosphate crystals, of ankle and foot | 67360 | N021700 |
| Chondrocalcinosis-pyrophosphate crystals, of multiple sites | 70575 | N021x00 |
| Chondrocalcinosis-pyrophosphate crystals, of other spec site | 66448 | N021y00 |
| Chondrocalcinosis-pyrophosphate crystals, of shoulder region | 64927 | N021100 |
| Chondrocalcinosis-pyrophosphate crystals, of the forearm | 36261 | N021300 |
| Chondrocalcinosis-pyrophosphate crystals, of the hand | 70727 | N021400 |
| Chondrocalcinosis-pyrophosphate crystals, of the lower leg | 65008 | N021600 |
| Chondrocalcinosis-pyrophosphate crystals, of the upper arm | 106535 | N021200 |
| Chondrocalcinosis-pyrophosphate crystals, of unspec site | 70839 | N021000 |
| Chondrodysplasia | 22756 | PG4..00 |
| Chondrodysplasia calcificans congenita | 63146 | PG56011 |
| Chondrodysplasia NOS | 14917 | PG4z.00 |
| Chondrodysplasia punctata | 18193 | PG4C.00 |
| Chondrodysplasia, unspecified | 73905 | PG40.00 |
| Chondroectodermal dysplasia | 23559 | PG55.00 |
| Chondrolysis | 37519 | N33zB00 |
| Chondrolysis of the femoral head | 55672 | N080C00 |
| Chondromalacia NOS | 1706 | N33z200 |
| Chondromalacia patellae | 1166 | N074.00 |
| Cockayne syndrome | 36567 | PKy6100 |
| Congenital exostosis | 10164 | PG47.00 |
| Congenital hypothyroidism | 10097 | C03..00 |
| Congenital hypothyroidism NOS | 51481 | C03z.00 |
| Congenital hypothyroidism with diffuse goitre | 31612 | C03y000 |
| Congenital hypothyroidism without goitre | 93159 | C03y100 |
| Congenital malformation syndrome with short stature NOS | 73575 | PKy6z00 |
| Congenital malformation syndromes with short stature | 57953 | PKy6.00 |
| Constitutional dwarfism | 65402 | C1z4100 |
| Constitutional tall stature | 8129 | C130300 |
| Craniometaphyseal dysplasia | 65348 | PG5C.00 |
| Diaphyseal aclasis | 26314 | PG48.00 |
| Diastrophic dwarfism | 68937 | PG44000 |
| Disorder of glucosaminoglycan metabolism, unspecified | 52080 | C375X00 |
| Disorders due to slow fetal growth, low and high birthweight | 68518 | Q1...00 |
| Dubowitz syndrome | 57043 | PKy6600 |
| Dwarfism NEC | 34706 | C1z4.00 |
| Dwarfism NEC NOS | 66781 | C1z4z00 |
| Dyschondroplasia NOS | 73909 | PG42z00 |
| Dysplasia epiphysealis hemimelica | 50583 | PG49.00 |
| Epiphysitis NOS | 9870 | N32z100 |
| Familial chondrocalcinosis | 96794 | N024.00 |
| Fetal growth retardation NOS | 19575 | Q10z.00 |
| Fetal malnutrition, no mention light or small for gest age | 104765 | Q102.00 |
| Fetus small-for-dates with signs of malnutrition | 63290 | Q101.00 |
| Fetus small-for-dates, without mention of malnutrition | 22032 | Q100.00 |
| Fibrochondrogenesis | 94825 | PG4B200 |
| Fragilitas ossium | 5486 | PG51000 |
| Goitrous cretin | 47449 | C031.00 |
| Haglund's disease - osteochondrosis of os tibiale externum | 25998 | N325500 |
| Hormone-induced hypopituitarism | 105672 | C137000 |
| Hypochondrogenesis | 95041 | PG4B100 |
| Hypochondroplasia | - | PG41000 |
| Hypophosphataemia | 7902 | C353300 |
| Hypophosphatasia | 33347 | C353000 |
| Hypophosphatasia rickets | 22028 | C353100 |
| Hypothyroidism NOS | 3941 | C04z.00 |
| Hypothyroidism resulting from para-aminosalicylic acid | 15743 | C043000 |
| Hypothyroidism resulting from phenylbutazone | 97090 | C043100 |
| Hypothyroidism resulting from resorcinol | 94915 | C043200 |
| Iatrogenic hypothyroidism NOS | 38976 | C043z00 |
| Idiopathic panhypopituitarism | 48590 | C132000 |
| Immunodeficiency with short-limbed stature | 65617 | C395.00 |
| Iodine hypothyroidism | 34221 | C042.00 |
| Irradiation hypothyroidism | 11322 | C041000 |
| Isolated deficiency of growth hormone | 1048 | C133000 |
| Juvenile apophysitis NOS | 28194 | N326000 |
| Juvenile epiphysitis NOS | 18812 | N326100 |
| Juvenile osteochondritis NOS | 6975 | N326200 |
| Juvenile osteochondroses NOS | 94547 | N326z00 |
| Juvenile osteochondrosis NOS | 9691 | N326300 |
| Juvenile osteochondrosis of the foot NOS | 60589 | N325z00 |
| Juvenile osteochondrosis of the leg | 33667 | N324.00 |
| Juvenile osteochondrosis of the leg, NOS | 38898 | N324z00 |
| Juvenile osteochondrosis of the leg, unspecified | 24028 | N324000 |
| Juvenile osteochondrosis of the secondary patellar centre | 48242 | N324300 |
| Kienbock's disease of adults | 8047 | N32y100 |
| Kniest dysplasia | 45412 | PG44500 |
| Kohler's disease - osteochondrosis of primary patella centre | 22689 | N324100 |
| Larsen's syndrome | 36839 | PFy1.00 |
| Late effect of rickets | 61243 | C281.00 |
| Lei-Weill dyschondrosteosis | 40661 | PG4F.00 |
| Lethal retarded ossification syndromes | 92892 | PG4B.00 |
| Mannosidosis | 107648 | C375000 |
| Mesomelic dysplasia | 58771 | PG44300 |
| Metachondromatosis | 70627 | PG4A.00 |
| Metaphyseal chondrodysplasia | 33811 | PG4D.00 |
| Metaphyseal dysostosis | 26069 | PG45.00 |
| Metatropic dwarfism | 37743 | PG44100 |
| Mild protein energy malnutrition | 57410 | C233.00 |
| Mild protein-calorie malnutrition (wt for age 75-89%) | 67272 | C231.00 |
| Moderate protein energy malnutrition | 36455 | C234.00 |
| Moderate protein-calorie malnutrition (wt for age 60-74%) | 98680 | C230.00 |
| Mucopolysaccharidosis | 22851 | C375.00 |
| Mucopolysaccharidosis NOS | 104999 | C375z00 |
| Mucopolysaccharidosis, type 1 | 69381 | C375100 |
| Mucopolysaccharidosis, type II | 61650 | C375200 |
| Mucopolysaccharidosis, type III | 70439 | C375300 |
| Mucopolysaccharidosis, type IV | 103139 | C375400 |
| Mucopolysaccharidosis, type VI | 62762 | C375600 |
| Multiple enchondromata | 35820 | PG42.00 |
| Multiple enchondromata with haemangioma | 59737 | PG42000 |
| Multiple epiphyseal dysplasia | 34387 | PG56.00 |
| Multiple epiphyseal dysplasia NOS | 101702 | PG56z00 |
| Multiple sulphatase deficiency | 72816 | C375800 |
| Myasthenic syndrome due to hypothyroidism | 61069 | F381400 |
| Myotonic chondrodysplasia | 105696 | PG42100 |
| Neonatal jaundice with congenital hypothyroidism | 58833 | Q433700 |
| Nutritional marasmus | 5247 | C21..00 |
| Osgood-Schlatter's dis - osteochondrosis of tibial tubercle | 1604 | N324400 |
| Osteochondritis dissecans | 3156 | N327.00 |
| Osteochondritis dissecans of lateral femoral condyle | 38335 | N327100 |
| Osteochondritis dissecans of other site | 42068 | N327y00 |
| Osteochondritis dissecans of patella | 54563 | N327000 |
| Osteochondritis dissecans of the capitellum | 70914 | N327400 |
| Osteochondritis dissecans of the femoral head | 34206 | N327800 |
| Osteochondritis dissecans of the humeral head | 103515 | N327300 |
| Osteochondritis dissecans of the radial head | 97447 | N327500 |
| Osteochondritis dissecans of the wrist | 97466 | N327700 |
| Osteochondritis NOS | 10122 | N32z200 |
| Osteochondrodyspl with defct growth tub bone spine unspec | 63565 | PGW..00 |
| Osteochondropathy NOS | 22861 | N32zz00 |
| Osteochondrosis NOS | 17135 | N32z300 |
| Osteogenesis imperfecta | 4158 | PG51.00 |
| Osteogenesis imperfecta - unclassifiable | 103017 | PG51200 |
| Osteogenesis imperfecta NOS | 53933 | PG51z00 |
| Osteogenesis imperfecta type I | 58635 | PG51300 |
| Osteogenesis imperfecta type II | 98662 | PG51400 |
| Osteogenesis imperfecta type III | 69436 | PG51500 |
| Osteogenesis imperfecta type IV | 97751 | PG51600 |
| Osteopathy/chondropathy/acquired musculoskeletal deformity | 37298 | N3...00 |
| Osteopsathyrosis | 46402 | PG51100 |
| Other acquired hypothyroidism | 24748 | C04y.00 |
| Other dwarfing syndromes NOS | 95921 | PG44z00 |
| Other iatrogenic hypothyroidism | 25913 | C043.00 |
| Other juvenile osteochondroses | 50425 | N326.00 |
| Other osteochondritis dissecans of knee | 35150 | N327200 |
| Other osteochondritis dissecans of the elbow | 60712 | N327600 |
| Other postablative hypothyroidism | 50275 | C041.00 |
| Other protein-calorie malnutrition | 59885 | C23y.00 |
| Other severe protein-calorie malnutrition (wt for age < 60%) | 26366 | C22..00 |
| Other specified congenital hypothyroidism | 69290 | C03y.00 |
| Other specified dwarfing syndromes | 65104 | PG44.00 |
| Other specified dwarfism NEC | 92129 | C1z4y00 |
| Other specified forms of osteochondropathy | 64064 | N32y.00 |
| Other specified osteochondropathy NOS | 62015 | N32yz00 |
| Other specified panhypopituitarism | 67154 | C132y00 |
| Other specified pituitary dwarfism | 69903 | C133y00 |
| Other+unsp protein-calorie malnutrition (wt for age 60-89%) | 54476 | C23..00 |
| Panhypopituitarism | 5026 | C132.00 |
| Panhypopituitarism NOS | 33653 | C132z00 |
| Pendred's syndrome | 11892 | C030.00 |
| Physical retardation due to protein-calorie malnutrition | 94237 | C232.00 |
| Pituitary dwarfism | 20287 | C133.00 |
| Pituitary dwarfism NOS | 61409 | C133z00 |
| Postablative hypothyroidism NOS | 51706 | C041z00 |
| Post-birth injury panhypopituitarism | 101601 | C132100 |
| Post-hypophysectomy hypopituitarism | 44881 | C137100 |
| Postinfarction panhypopituitarism | 70695 | C132200 |
| Postinfectious hypothyroidism | 50860 | C044.00 |
| Postinfective panhypopituitarism | 44873 | C132300 |
| Post-radiotherapy hypopituitarism | 44247 | C137200 |
| Postsurgical hypothyroidism | 28852 | C040.00 |
| Precocious puberty with adrenocortical hyperfunction | 7018 | C152900 |
| Premature puberty due to hypothyroidism | 56722 | C04z000 |
| Protein malnutrition unspecified | 38024 | C20z.00 |
| Protein-calorie malnutrition NOS | 36951 | C23z.00 |
| Pseudoachondroplasia | - | PG446 |
| Psychosocial dwarfism | 93846 | C1z4000 |
| Renal rickets | 66062 | K080300 |
| Robinow syndrome | 57042 | PKy6700 |
| Russell - Silver syndrome | 32868 | PKy6200 |
| Seckel syndrome | 34719 | PKy6400 |
| Short-rib/polydactyly syndrome | 18160 | PG4B300 |
| Slow fetal growth and fetal malnutrition | 34777 | Q10..00 |
| Smith - Lemli - Opitz syndrome | 33522 | PKy6300 |
| Spondyloepiphyseal dysplasia | 27802 | PG46.00 |
| Spondyloepiphyseal dysplasia congenita | 100727 | PG46000 |
| Spondyloepiphyseal dysplasia tarda | 93058 | PG46100 |
| Spondylometaphyseal dysplasia | 48184 | PG4E.00 |
| Subclinical iodine-deficiency hypothyroidism | 718 | C0A5.00 |
| Synovial osteochondromatosis | 16411 | N098.00 |
| Synovial osteochondromatosis of acromioclavicular joint | 107105 | N098200 |
| Synovial osteochondromatosis of ankle | 94369 | N098D00 |
| Synovial osteochondromatosis of elbow | 17655 | N098300 |
| Synovial osteochondromatosis of hip | 41852 | N098900 |
| Synovial osteochondromatosis of knee | 45749 | N098B00 |
| Synovial osteochondromatosis of lesser MTP joint | 99679 | N098J00 |
| Synovial osteochondromatosis of MCP joint | 104827 | N098600 |
| Synovial osteochondromatosis of other tarsal joint | 104882 | N098G00 |
| Synovial osteochondromatosis of PIP joint of finger | 101839 | N098700 |
| Synovial osteochondromatosis of shoulder | 101957 | N098000 |
| Synovial osteochondromatosis of talonavicular joint | 106077 | N098F00 |
| Synovial osteochondromatosis of wrist | 106485 | N098500 |
| Thanatophoric dwarfism | 56900 | PG44200 |
| Trichorhinophalangeal syndrome | 36477 | PKy5A00 |
| Turner's phenotype, karyotype 45X | 30721 | PJ63100 |
| Turner's phenotype, karyotype 46X iso (Xq) | 92599 | PJ63200 |
| Turner's phenotype, karyotype normal | 98244 | PJ63000 |
| Turner's phenotype, mosaicism 45X/46XX or 45X/46XY | 51868 | PJ63400 |
| Turner's phenotype, other variant karyotypes | 65206 | PJ63600 |
| Turner's syndrome | 4943 | PJ63.00 |
| Turner's syndrome NOS | 53168 | PJ63z00 |
| Turner's,karyotype 46X + abnorm. sex chromosome,not iso(Xq) | 109385 | PJ63300 |
| Turner's,mosaic, 45X/other cell line with abn.sex chromosome | 40570 | PJ63500 |
| Unspecified severe protein-energy malnutrition | 55008 | C20zX00 |
| Vitamin-D-resistant rickets | 33586 | C353200 |

**Abbreviations:** MCP: metacarpophalangeal; NOS: not otherwise specified; PIP: Proximal Interphalangeal.
