# Supplementary material for: Higher rates of non-skeletal complications and greater healthcare needs in achondroplasia compared to the general UK population: a matched cohort study using the CPRD database
Source: Orphanet J Rare Dis. 2023 Jul 25;18:211. doi: 10.1186/s13023-023-02811-5 (PMC10367327; doi:10.1186/s13023-023-02811-5)
Supplement: Supplementary file 4 — Additional file 4. Outcomes by age group among ACH cases (CPRD and CPRD HES-linked cohorts). [file 13023_2023_2811_MOESM4_ESM.docx]

Additional File 4: Outcomes by age group among ACH cases (CPRD and CPRD HES-linked cohorts)

| **By body system category and age group** | | | | | **Specific events by age group*** | | |
| --- | --- | --- | --- | --- | --- | --- | --- |
| **Category** | **n, ER per 100PY**** | | | | **Specific event** | **n, ER per 100PY**** | |
|  | **0-10 years** | **11-17 years** | **18-59 years** | **≥60 years** |  | **< 18 years** | **≥18 years** |
| **Hospital surgical procedures (CPRD HES-linked cohort)***** | | | | |  |  |  |
| Any procedure | 113, 16.59 | 44, 9.40 | 88, 2.64 | 20, 3.02 | - | - | - |
| ENT | 73, 10.72 | 12, 2.56 | 17, 0.51 | <5, NC | Grommet insertion | 58, 5.05 | 8, 0.20 |
|  |  |  |  |  | Hearing aid | <5, 0.09 | 5, 0.13 |
|  |  |  |  |  | Tonsillectomy/adenoidectomy | 21, 1.83 | <5, NC |
|  |  |  |  |  | Tympanoplasty | 5, 0.44 | <5, NC |
| Neurological | <10, NC | 0 | <5, NC | 0 | Shunt/ventriculostomy | <10, NC | <5, NC |
| Orthopaedic | 33, 4.84 | 30, 6.41 | 65, 1.95 | 13, 1.96 | - | - | - |
| Limb*** | 24, 3.52 | 24, 5.13 | 16, 0.48 | <5, NC | Bone fixation (internal/external) | 39, 3.39 | 5, 0.13 |
|  |  |  |  |  | Joint replacement (hip/knee) | <5, NC | 10, 0.25 |
|  |  |  |  |  | Limb lengthening | 9, 0.78 | <5, 0.03 |
| Spinal | 9, 1.32 | 6, 1.28 | 49, 1.47 | <15, NC | Foramen magnum decompression | <5, NC | <5, NC |
|  |  |  |  |  | Spinal decompression | 11, 0.96 | 58, 1.45 |
| Respiratory | <5, NC | <5, NC | <5, NC | <5, NC | Ventilation | <5, NC | 7, 0.18 |
| **Medication use (CPRD cohort)** | | | | |  |  |  |
| Any Medication | 1284, 191.92 | 831, 203.86 | 24099, 817.63 | 22856, 2,651.37 | - | - | - |
| Antibiotics/antifungal | 875, 130.78 | 295, 72.37 | 2174, 73.76 | 1134, 131.55 | - | - | - |
| Cardiovascular | 0 | 0 | 3745, 127.06 | 7639, 886.15 | Anti-hypertensives | 0 | 2399, 119.16 |
|  |  |  |  |  | Cardiac | 0 | 467, 23.20 |
|  |  |  |  |  | Statins | 0 | 853, 42.37 |
| Digestive | 40, 5.98 | 41, 10.06 | 2205, 74.81 | 2554, 296.27 | Gastrointestinal | 8, 1.32 | 233, 11.57 |
|  |  |  |  |  | Proton pump inhibitors | 49, 8.10 | 1224, 60.80 |
| Genito-urinary | 16, 2.39 | 48, 11.78 | 946, 32.10 | 721, 83.64 | Urinary incontinence | 16, 2.64 | 726, 36.06 |
| Haematological | 0 | 26, 6.38 | 87, 2.95 | 315, 36.54 | Anticoagulants | 7, 1.16 | 49, 2.43 |
| Metabolism and Endocrine | 0 | 11, 2.70 | 839, 28.47 | 953, 110.55 | Anti-obesity | 0 | 69, 3.43 |
|  |  |  |  |  | Diabetes | 11, 1.82 | 419, 20.81 |
| Musculoskeletal | 336, 50.22 | 238, 58.39 | 9201, 312.17 | 6077, 704.95 | Bone disorders | 0 | 913, 45.35 |
|  |  |  |  |  | Pain | 218, 36.03 | 5432, 269.82 |
| Nervous | 17, 2.54 | 172, 42.20 | 4902, 166.31 | 3463, 401.72 | Anti-epileptics | 10, 1.65 | 559, 27.77 |
|  |  |  |  |  | Antidepressants/anxiolytics | 10, 1.65 | 2019, 100.29 |
|  |  |  |  |  | ADHD | 51, 8.43 | 0 |
|  |  |  |  |  | Dementia | 0 | 77, 3.82 |
|  |  |  |  |  | Substance dependence | 0 | 50, 2.48 |

*Due to small cell sizes, specific events by age group are presented within broader <18 or ≥18 year age categories

**Where the number of events in a cell was less than 5, this has been stated as <5 due to CPRD reporting requirements. In some cases, values greater than 5 have been reported as less than (< the next highest multiple of 5) to prevent calculation of exact values elsewhere.

***Data on cardiac surgeries and brace provisions have not been reported by age group, since the overall number of events was <5.

Abbreviations: ACH, achondroplasia; ADHD, attention deficit hyperactivity disorder; ; CPRD, Clinical Practice Research Database; ENT, ear, nose and throat; ER, event rate; n, number of events of interest; NC, not calculated (due to small cell size); PY, person years.
