# Supplementary material for: Higher rates of non-skeletal complications and greater healthcare needs in achondroplasia compared to the general UK population: a matched cohort study using the CPRD database
Source: Orphanet J Rare Dis. 2023 Jul 25;18:211. doi: 10.1186/s13023-023-02811-5 (PMC10367327; doi:10.1186/s13023-023-02811-5)
Supplement: Supplementary file 5 — Additional file 5. Rate of GP referrals to specialist care among cases and controls (CPRD cohort). [file 13023_2023_2811_MOESM5_ESM.docx]

Additional File 5: Rate of GP referrals to specialist care among cases and controls (CPRD cohort)

| **Referral type** | **n, ER per 100PY** | | **RR (95% CI)** |
| --- | --- | --- | --- |
|  | **Cases (N=541)** | **Controls (N=2052)** |  |
| Any referral | 979, 20.04 | 2550, 10.57 | 1.93 (1.66–2.24)* |
| Occupational therapy | 15, 0.31 | 16, 0.07 | 4.78 (2.31–9.89)* |
| Wheelchair referral | 5, 0.10 | <5, NC | NC |
| Dietician/weight management | 19, 0.39 | 25, 0.10 | 3.76 (1.88–7.50)* |
| Neurology/neurosurgery | 33, 0.68 | 44, 0.18 | 3.71 (2.08–6.60)* |
| Dental/maxillofacial | 8, 0.16 | 11, 0.05 | 3.58 (1.35–9.49)* |
| Nurse/outpatient | 58, 1.19 | 91, 0.38 | 3.26 (1.53–6.97)* |
| Pain management | 14, 0.29 | 22, 0.09 | 3.14 (1.14–8.66)* |
| Orthopaedics | 103, 2.11 | 185, 0.77 | 2.84 (2.13–3.79)* |
| Respiratory/sleep clinic | 19, 0.39 | 34, 0.14 | 2.81 (1.41–5.61)* |
| Endocrinology/diabetes management | 6, 0.12 | 12, 0.05 | 2.47 (0.88–6.93) |
| ENT | 78, 1.60 | 179, 0.74 | 2.12 (1.58–2.82)* |
| Physiotherapy | 83, 1.70 | 196, 0.81 | 2.10 (1.49–2.95)* |
| Accident & emergency | 13, 0.27 | 31, 0.13 | 2.05 (0.76–5.50) |
| Paediatrics | 30, 0.61 | 71, 0.29 | 2.01 (1.20–3.36)* |
| Ophthalmology | 39, 0.80 | 98, 0.41 | 1.96 (1.24–3.08)* |
| Specialist imaging | 84, 1.72 | 217, 0.90 | 1.90 (1.30–2.79)* |
| Gastroenterology | 26, 0.53 | 75, 0.31 | 1.77 (1.11–2.82)* |
| Urology/incontinence | 19, 0.39 | 67, 0.28 | 1.40 (0.72–2.71) |
| General medicine | 186, 3.81 | 661, 2.74 | 1.39 (1.09–1.76)* |
| Dermatology | 37, 0.76 | 135, 0.56 | 1.37 (0.89–2.11) |
| General surgery | 45, 0.92 | 164, 0.68 | 1.36 (0.86–2.15) |
| Mental health/counselling/substance abuse | 32, 0.65 | 123, 0.51 | 1.34 (0.82–2.18) |
| Cardiology | 23, 0.47 | 90, 0.37 | 1.24 (0.70–2.20) |
| Geneticist | <5, NC | <5, NC | NC |
| Speech therapy | <5, NC | <5, NC | NC |

*Statistically significant result (a=0.05).

**Abbreviations:** CPRD, Clinical Practice Research Database; ENT, ear, nose and throat; GP, general practitioner; N, total number of individuals; NC: not calculated (due to small cell size); PY, person years; RR, rate ratio.
