# Supplementary material for: Higher rates of non-skeletal complications and greater healthcare needs in achondroplasia compared to the general UK population: a matched cohort study using the CPRD database
Source: Orphanet J Rare Dis. 2023 Jul 25;18:211. doi: 10.1186/s13023-023-02811-5 (PMC10367327; doi:10.1186/s13023-023-02811-5)
Supplement: Supplementary file 6 — Additional file 6. Underlying cause of death by ICD-10 chapter among ACH cases vs controls (CPRD HES-linked cohort). [file 13023_2023_2811_MOESM6_ESM.docx]

**Additional File 6: Underlying cause of death by ICD-10 chapter among ACH cases vs controls (CPRD HES-linked cohort)**

| **Underlying cause of death (ICD-10 chapter), n (% of total deaths)*** | **Cases (N=541)** | **Controls (N=2052)** |
| --- | --- | --- |
| All reported deaths | 31 (100) | 69 (100) |
| Diseases of the circulatory system [I00-I99] | 7 (23) | 24 (35) |
| Neoplasms [C00-D49] | 5 (16) | 19 (28) |
| Congenital malformations, deformations and chromosomal abnormalities [Q00-Q99] | 5 (16) | 0 (0) |
| Diseases of the respiratory system [J00-J99] | <5 (NC) | 7 (10) |
| Diseases of the digestive system [K00-K95] | <5 (NC) | 8 (12) |
| Diseases of the genitourinary system [N00-N99] | <5 (NC) | <5 (NC) |
| Mental/behavioural/neurodevelopmental disorders [F00-F99] and nervous system [G00-99] | <5 (NC) | <5 (NC) |
| Diseases of skin/subcutaneous tissue; musculoskeletal system/connective tissue | <5 (NC) | 0 (0) |
| Injury/poisoning/other external causes [S00-S99; V01-Y98] | <5 (NC) | <5 (NC) |
| Symptoms, signs and abnormal clinical and laboratory findings, not elsewhere classified | 0 (0) | <5 (NC) |
| Unknown | <5 (NC) | 6 (9) |

*Where the number of patients was reported as an integer of more than 0 but less than 5, this has been stated as <5 due to CPRD reporting requirements.

Abbreviations: ACH, achondroplasia; CPRD, Clinical Practice Research Database; HES, Hospital Episode Statistics ICD-10: International Classification of Diseases 10th Revision; N, total number of individuals; n, number of individuals in subset; NC, not calculated (due to small cell size).
